# DBSsync: combining intracranial and multimodal data to investigate new biomarkers in Parkinson’s disease

**DOI:** 10.1038/s41531-026-01439-z

**Published:** 2026-06-19

**Authors:** Juliette Vivien, Charlotte E. Stensholt, Lucie Hortmann, Merle Hendel, Arian Memarpouri, Roxanne Lofredi, Jeroen G. V. Habets, Alessia Cavallo, Lucia K. Feldmann, Andrea A. Kühn

**Affiliations:** 1 Department of Neurology, Charité-Universitätsmedizin Berlin, Berlin, Germany; 2 Humboldt-Universität zu Berlin, Berlin School of Mind and Brain, Berlin, Germany; 3 Berlin Institute of Health (BIH), Berlin, Germany; 4 Bernstein Center for Computational Neuroscience, Humboldt-Universität, Berlin, Germany; 5 NeuroCure, Exzellenzcluster, Charité-Universitätsmedizin Berlin, Berlin, Germany; 6 DZNE, German Center for Neurodegenerative Diseases, Berlin, Germany

**Keywords:** Biological techniques, Biomarkers, Engineering, Neurology, Neuroscience

## Abstract

Implanted deep brain stimulation devices are now capable of chronically recording activity from intracranial brain areas during stimulation. This new type of data has the potential to increase our understanding of disease-related brain activity and its modulation in response to therapy or other types of stimuli. With the innovative approach of adaptive deep brain stimulation now clinically available, multimodal characterization of neural biomarkers becomes of utmost importance to define optimal feedback signals for adaptive brain stimulation and allow for better fine-tuning of stimulation parameters. To investigate these biomarkers, we developed DBSsync, a paradigm and an open-source Python toolbox with its graphical user interface for temporally precise synchronization of intracranial recordings with external data, allowing for multimodal research protocols. DBSsync achieves a temporal precision of 8 ms and incorporates cardiac artifact removal methods to facilitate intracranial data preprocessing, thus enabling the integration and precise synchronization of multiple brain signals with external sensors and various behavioral timeline data.

## Introduction

Deep brain stimulation (DBS) is an established and effective treatment for severe movement disorders^1,2^. Recently, DBS devices with sensing capacities have become commercially available, widening research horizons by making intracranial electrophysiological recordings accessible to a growing research field. This new feature allows for high-quality local field potential (LFP) recordings in chronically implanted patients, outside of the peri-operative period, therefore with more stable electrophysiology, clinical recovery, and optimized therapy. This has led to the discovery of new modulations from known biomarkers, such as diurnal modulation of beta power^3^ in patients with Parkinson’s disease (PD). New biomarkers were also discovered, such as stimulation-entrained gamma for motor improvement^4,5^ and beta-gamma phase-amplitude coupling related to gait^6^. Discovering personalized neural biomarkers is also important for adaptive DBS (aDBS), as Louie and colleagues have described in five patients treated with aDBS timed to neural biomarkers of contralateral leg swing. Their approach resulted in improved gait compared to continuous DBS treatment^7^. With the rise of these aDBS protocols for better management of both motor and non-motor symptoms in PD^8,9^, precise investigation and validation of specific biomarkers become even more essential to decipher complex pathophysiological mechanisms involving multiple brain areas and their link to behavioral readouts. For such a multimodal research approach, the integration and synchronization of multiple brain signals with external sensors and various behavioral timeline data is necessary.

Intracranial recordings via sensing-enabled DBS devices are currently transmitted via Bluetooth to a tablet programmed for clinical use. A common connection to a specific recording device that would allow for parallel acquisition of data from additional external sensors, such as accelerometers or electroencephalogram (EEG), is therefore not possible. The detection of a shared signal in several recording modalities is needed to perform such synchronization. For example, delivering short DBS pulses can generate artifacts in intracranial recordings that are also detectable near the Percept^TM^ (Percept^TM^ PC/RC, Medtronic, Minneapolis, MN, USA) implantable pulse generator (IPG)^10^. To this date, the reliability and reproducibility of DBS artifacts across patients remain unclear and no open-source and ready-to-use software relying on DBS artifacts for automatic synchronization with multimodal data is yet available. A recent paper^11^ proposed a method and toolbox for the synchronization of DBS data with EEG recordings and used the switch-on artifact of the Percept^TM^ device. However, this toolbox is limited to EEG recordings, not considering data obtained from wearables, videos or other sources that would complete the clinical/behavioral picture during assessment. Additionally, recordings from sensing-enabled deep brain stimulators are often contaminated by electrocardiogram (ECG) artifacts and there is a need for the implementation of reliable and easy-to-use ECG suppression methods^12,13^.

Here, we describe our open-source Python toolbox called “DBSsync”, offering precise synchronization between chronic intracranial recordings via sensing-enabled DBS devices (Medtronic Percept^TM^) and external recordings of various sources, such as wearables, videos or task-related events, validated in 25 patients with PD and bilaterally implanted with DBS electrodes in the subthalamic nucleus (STN). DBSsync provides an easy-to-use Graphical User Interface (GUI) with multiple features such as ECG artifact cleaning, correction of the Percept^TM^ sampling frequency and verification of proper alignment of the signals after long recording durations.

## Results

**Fig. 1:**
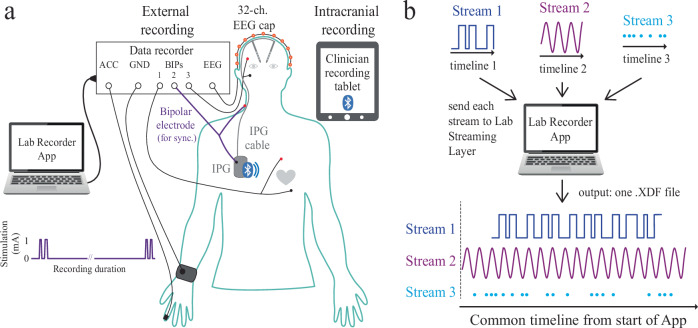
Recording setup and synchronization of multiple external streams. **a** Example of setup for dataset 2: the patient is equipped with three bipolar electrodes (“BIPs”): one is used for detecting DBS synchronization pulses and is placed close to the Implantable Pulse Generator (IPG) and cable, another is recording eye movements (EOG channel) and the last is recording the heartbeat (ECG channel). An accelerometer (ACC) is placed on the index finger to record button presses during a behavioral task, and an electroencephalography (EEG) cap with 32 channels is also used to record cortical electrophysiological activity. All these channels are recorded via an external digital amplifier, which is streamed to the Lab Recorder Application. The activity from the STN is recorded from implanted DBS electrodes and sent via Bluetooth connection to the clinician recording tablet. Two synchronization DBS pulses are performed at the beginning and at the end of each recording session. GND: ground. **b** The Lab Recorder application can receive streams from different sources (external digital amplifier, behavioral task, video camera, etc.). It automatically synchronizes all streams and, at the end of the recording session, saves one single output file in .XDF format, with a common timeline.

Figure 1 shows an example setup and system for the acquisition and synchronization of multimodal data with chronic Percept-LFP data using stimulation pulses and lab streaming layer (LSL). The results presented below are based on three different test datasets, each containing multiple recording sessions (for details, see “Methods” section and Table 1).

**Table 1:** Overview of participants and session features of each dataset

Dataset	Session	Time since surgery	Digital amplifier sampling frequency	Synchronization pulse features	STN recording contacts	Cardiac artifact in the LFP?
1	sub021 M1S1	24MFU	4000Hz	LSTN: 1mA, 125Hz, 60µs. Contact 1b	LSTN: 0-2	N
					RSTN: 0-2	Y^β^
	sub024 M0S1	24MFU	4096Hz	LSTN: 1mA, 85Hz, 40µs. Contact 1	LSTN: 0-2	N
					RSTN: 0-2	Y^β^
	sub033 M1S0	24MFU	4096Hz	LSTN: 1mA, 125Hz, 60µs. Contact 2	LSTN: 1-3	Y^β^
					RSTN: 1-3	Y^β^
	sub048 M0S1	18MFU	4000Hz	LSTN: 1mA, 125Hz, 40µs. Contact 2	LSTN: 1-3	Y
					RSTN: 0-2	Y
	sub051 M1S1	18MFU	4000Hz	LSTN: 1mA, 125Hz, 60µs. Contact 1	LSTN: 0-2	N
					RSTN: 0-2	N
	sub059 M0S0	12MFU	4000Hz	LSTN: 1mA, 125Hz, 60µs. Contact 1	LSTN: 0-2	N
					RSTN: 1-3	N
	sub061 M0S1	12MFU	4096Hz	LSTN: 1mA, 85Hz, 60µs. Contact 2	LSTN: 1-3	Y
					RSTN: 1-3	N
	sub067 M1S0	3MFU	4000Hz	LSTN: 1mA, 125Hz, 60µs. Contact 1	LSTN: 0-2	N
					RSTN: 0-2	N
	sub070 M1S0	3MFU	512Hz	LSTN: 1mA, 145Hz, 60µs. Contact 2	LSTN: 1-3	Y
					RSTN: 1-3	Y
	sub084 M1S0	3MFU	2048Hz	LSTN: 1mA, 125Hz, 60µs. Contact 1	LSTN: 0-2	Y
					RSTN: 1-3	N
	sub069 M1S0	12MFU	4096Hz	LSTN: 1mA, 125Hz, 40µs. Contact 2	LSTN: 1-3	N
					RSTN: 1-3	N
2	sub083 M1S1	22MFU	4096Hz	LSTN: 1mA, 110Hz, 60µs. Contact 1	LSTN: 0-2	N
					RSTN: 0-2	Y
	sub021 M1S1	38MFU	2048Hz	LSTN: 1mA, 125Hz, 60µs. Contact 1b	LSTN: 0-2	N
					RSTN: 0-2	N
	sub065 M1S1	17MFU	2048Hz	LSTN: 1mA, 125Hz, 60µs. Contact 2	LSTN: 1-3	Y*
					RSTN: 0-2	Y
	sub065 M1S0	17MFU	2048Hz	LSTN: 1mA, 125Hz, 60µs. Contact 2	LSTN: 1-3	Y*
					RSTN: 0-2	Y^β^
	sub050 M1S1	30MFU	2048Hz	LSTN: 1mA, 180Hz, 60µs. Contact 2	LSTN: 1-3	Y
					RSTN: 1-3	Y
	sub096 M1S1	10MFU	2048Hz	LSTN: 1mA, 125Hz, 60µs. Contact 2	LSTN: 1-3	N
					RSTN: 1-3	N
	sub052 M1S0	27MFU	2048Hz	LSTN: 1mA, 145Hz, 60µs. Contact 2	LSTN: 1-3	Y^β^
					RSTN: 0-3	N
	sub047 M1S1	29MFU	2048Hz	LSTN: 1mA, 125Hz, 60µs. Contact 2	LSTN: 1-3	Y*
					RSTN: 0-2	Y
	sub047 M1S0	29MFU	2048Hz	LSTN: 1mA, 125Hz, 60µs. Contact 2	LSTN: 1-3	Y*
					RSTN: 0-2	Y
	sub066 M1S0	21MFU	2048Hz	LSTN: 1mA, 125Hz, 60µs. Contact 1	LSTN: 0-2	N
					RSTN: 0-2	Y^β^
	sub122 M1S0	7MFU	2048Hz	LSTN: 1mA, 125Hz, 60µs. Contact 2	LSTN: 1-3	N
					RSTN: 1-3	N
	sub078 M1S1	18MFU	2048Hz	LSTN: 1mA, 125Hz, 60µs. Contact 1	LSTN: 0-2	Y*
					RSTN: 0-2	N
	sub078 M1S0	18MFU	2048Hz	LSTN: 1mA, 125Hz, 60µs. Contact 1	LSTN: 0-2	Y*
					RSTN: 0-2	Y
	sub080 M1S0	17MFU	2048Hz	LSTN: 1mA, 125Hz, 60µs. Contact 1-2	LSTN: 0-3	N
					RSTN: 0-2	N
	sub070 M1S1	21MFU	2048Hz	LSTN: 1mA, 145Hz, 60µs. Contact 2	LSTN: 1-3	Y*
					RSTN: 1-3	Y
	sub093 M1S1	15MFU	2048Hz	LSTN: 1mA, 125Hz, 60µs. Contact 2	LSTN: 1-3	Y
					RSTN: 1-3	Y*
	sub043 M1S1	34MFU	2048Hz	LSTN: 1mA, 125Hz, 60µs. Contact 1	LSTN: 0-2	N
					RSTN: 0-2	N
	sub108 M1S0	10MFU	2048Hz	LSTN: 1mA, 85Hz, 60µs. Contact 2	LSTN: 1-3	N
					RSTN: 1-3	N
3	sub051 M1S0	36MFU	4000Hz	LSTN: 1mA, 125Hz, 60µs. Contact 1	LSTN: 0-2	N
					RSTN: 0-2	N
	sub051 M1S1	36MFU	4000Hz	LSTN: 1mA, 125Hz, 60µs. Contact 1	LSTN: 0-2	N
					RSTN: 0-2	N
	sub064 M1S0	24MFU	4000Hz	LSTN: 1mA, 125Hz, 60µs. Contact 1	LSTN: 0-2	N
					RSTN: 1-3	N
	sub080 M1S0	24MFU	4000Hz	LSTN: 1mA, 125Hz, 60µs. Contact 1	LSTN: 0-2	N
					RSTN: 0-2	N
	sub080 M1S1	24MFU	4000Hz	LSTN: 1mA, 125Hz, 60µs. Contact 1	LSTN: 0-2	N
					RSTN: 0-2	N

### Stimulation artifacts are reproducible across patients

**Fig. 2:**
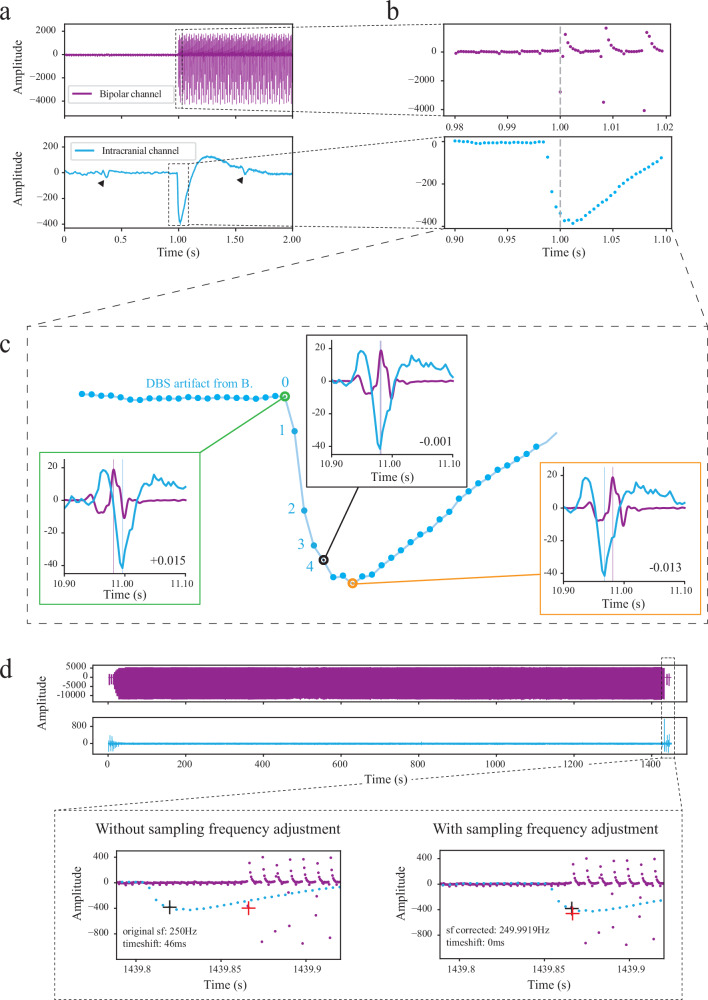
Synchronization of intracranial and external recordings. **a** In the external bipolar electrode, a DBS synchronization pulse induces a fast and repetitive deflection in the amplitude (top plot), whereas in the intracranial channel it induces a sharp drop in the amplitude of the signal, followed by a slow recovery (lower plot). **b** Zoom on (**a**), gray dashed lines show which sample is chosen as start of the artifact in each recording modality for the synchronization in DBSsync. **c** Method used to investigate which sample of the DBS artifact to choose as “start of the artifact” in the Percept-LFP timeseries. The blue dotted line represents a typical artifact shape as seen in the intracranial channel from (**b**). The last sample before the sharp change in amplitude is labeled as sample 0. Different samples were chosen for the synchronization to test which sample provides the best alignment of endogenous cardiac artifacts between the intracranial channel and an externally acquired ECG channel. As depicted in this example, sample number 4 provided the best results, here with a delay of −1 ms observed between the two cardiac artifacts. This sample is always chosen for synchronization in the toolbox, as depicted in (**b**). **d** Full recording session with external bipolar electrode (top) and intracranial (bottom) channels synchronized. The two synchronization pulses at the end (dashed box) are used to calculate the timeshift between the two recordings. After more than 1400 s of recording, as depicted on the lower plots, the last synchronization pulse is visible with a delay of 46 ms in the two synchronized channels (left). After a correction of the sampling frequency (sf) of the Percept-LFP data, the synchronization pulse is now perfectly aligned at the end of the two channels (right).

In order to generate synchronization pulses, two abrupt changes of stimulation amplitude are performed at the beginning and at the end of each recording session by sliding the amplitude up and down quickly (Fig. 1a, see “Methods” for detailed protocol). These changes in DBS amplitude gave rise to easily identifiable artifacts in both the Percept-LFP data and in an externally recorded bipolar channel placed around the IPG and the cable (Fig. 2a). In intracranial recordings, the Percept^TM^ hardware suppresses DBS artifacts after a first visible deflection in the signal. In the external bipolar electrode, the artifact is continuously recorded when DBS is turned on and is seen as a fast, repetitive change in signal amplitude, matching the stimulation frequency (Fig. 2a, b). While the artifact shape in intracranial recordings can be slightly different, it is stable in external recordings even with various sampling frequencies (2048 Hz, 4 kHz, 4096 Hz). In both signals the polarity of the artifact can vary, but this is accounted for automatically in DBSsync (correct detection of polarity: 100% in both signal types).

### DBSsync enables automatic and reliable synchronization of multimodal data

Out of 34 recording sessions, automatic detection of the synchronization artifact was successful in 34 external bipolar channels (100%) and in 33 Percept-LFP channels (97%) (see Fig. 2b for an example of correct artifact detection). In the remaining Percept-LFP recording, the start of the artifact timepoint was detected two samples too early and was therefore adjusted manually. As described in the methods section, cardiac artifacts were used to determine temporal accuracy of our synchronization method (Fig. 2c). Using the best sample for synchronization in 8 independent STN (i.e., not recorded during the same session), cardiac artifacts after 10 s of recording were never shifted by more than 6 ms between Percept-LFP and external ECG, therefore achieving a temporal resolution of 8 ms (6 ms corresponding to a difference of 1–2 samples in the Percept-LFP data sampled at 250 Hz).

### DBSsync helps detect and correct timescale inaccuracies

As DBS pulses were repeated at the end of each recording session, the timestamps of the last artifact in the intracranial and external recordings were systematically compared after synchronization to assess data integrity and potential timeshift. As described in the methods section, timeshift may arise after longer recording duration due to small variations in the sampling frequency of the Percept^TM^ device around the 250 Hz value indicated and, in less common cases, from the result of packet loss. DBSsync automatically detects packet loss when the .JSON file is directly imported, and adds NaN values in place of the missing data. Out of 34 recording sessions, 3 contained packet loss and were flagged by DBSsync as “corrected for packet loss”. Positive or negative timeshift values were found in all recordings (mean ± std: 11 ± 26 ms). For all recording sessions, the *effective sampling frequency* was computed and varied between 249.985 Hz and 250.024 Hz (mean ± std: 249.997 ± 0.008 Hz), which is a reasonable range given the small variations expected. Therefore, this computed *effective sampling frequency* was used to create a more accurate timescale for Percept-LFP recordings and synchronize it again with external data, ensuring higher accuracy and consistent alignment of the signals over longer periods of time. After this adjustment of the sampling frequency, timeshift at the end of each recording was computed again and was always close to 0 ms (Fig. 2d).

### DBSsync enables reliable cleaning of Percept-LFP data from cardiac artifacts

**Fig. 3:**
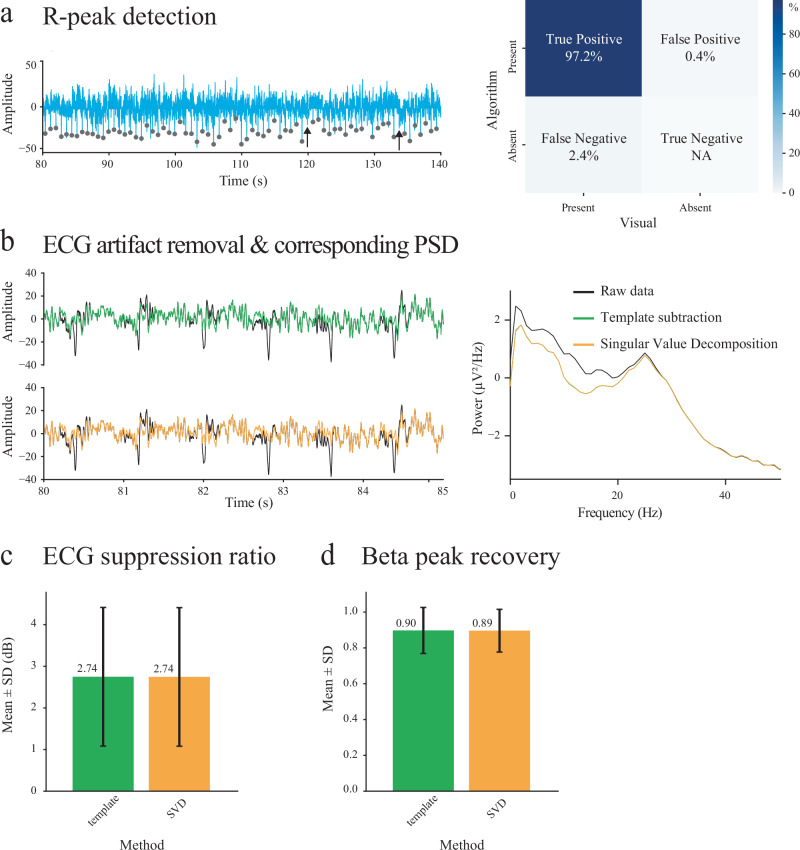
ECG artifact cleaning in Percept-LFP data. **a** R-peak detection and visual verification. R-peaks automatically detected in the Percept-LFP channel (in blue) by DBSsync are labeled with gray dots. In this example, two R-peaks were missed (identified with black arrows). This visual verification over 1 min of recording was performed in all analyzed sessions and overall DBSsync performance was calculated and reported in the confusion matrix on the right side. **b** ECG artifact removal using two different methods. Raw Percept-LFP time series (dark line) overlapped with cleaned data after using template subtraction (top) or Singular Value Decomposition (bottom) to remove ECG artifacts. The resulting power spectrum for each time series is shown on the right side. **c** ECG suppression ratio (in the 0.5–40 Hz frequency band) achieved by each cleaning method (*n* = 28 STN, mean ± SD). There were no significant differences between methods. **d** Beta peak recovery achieved by each cleaning method. Only prominent peaks in the 13–35 Hz frequency range were used (*n* = 7 STN, mean ± SD). There were no significant differences between methods.

30 out of 68 STN contained visible cardiac artifacts. For each of these recordings, R-peaks were automatically detected, and results were compared with visual verification across 1 min of recording (Fig. 3a). In 2 STN from dataset 1, R-peaks were of small amplitude compared to true brain signal and could not be detected properly because of the absence of an external ECG channel. These 2 STN recordings were therefore not included in subsequent analysis. For each of the 28 remaining STN the best possible method was used (either solely the Percept-LFP data or in combination with a synchronized external ECG channel when available). Across sessions, DBSsync detected overall 97.2% of true positive R-peaks, 0.4% of false positive and 2.4% of R-peaks were missed (Fig. 3a, see black arrows for missed peaks and gray dots for correctly detected R-peaks). Each of the two cleaning methods was then applied separately and an overlap of raw and cleaned signals was plotted in the GUI (Fig. 3b shows an example of the result for each method and their associated power spectra). Power spectra of raw and cleaned channels were also saved for subsequent analysis and evaluation of each cleaning method. A Wilcoxon signed-rank test was performed to compare the effect of these two different cleaning methods on ECG suppression ratio. The test revealed that there was no statistically significant difference in ECG suppression ratio between template subtraction and SVD1 methods. Beta peak recovery was assessed in 7 STN presenting a prominent beta peak. There was also no significant difference in beta peak recovery across methods, with both methods achieving values close to 1 indicating good preservation of beta oscillatory activity without spectral enhancement (Fig. 3d). Tests on synthetic signals confirmed that both the template subtraction and SVD methods achieved signal-to-noise ratio (SNR) improvement for both low and high contamination levels (−10 dB to +10 dB, Supplementary Fig. 4). Correlation between cleaned signals and uncontaminated synthetic signals was also high and above 90% for all methods up to 5 dB contamination (Supplementary Fig. 4). Synthetic theta and beta power were well preserved with beta power preservation (BPP) and theta power preservation (TPP) values close to 1. Of note, template subtraction and SVD1 method tend to enhance beta power in a SNR-dependent trend. This observation was expected because with higher contamination levels we also observed ECG-like SVD components in lower-ranked components more often. Therefore, we would recommend using the SVD method with more than one component when contamination level (SNR) is high (Supplementary Fig. 4).

## Discussion

In this paper, we present a method to perform temporally precise offline synchronization of intracranial with external recordings using our toolbox DBSsync. One of the major achievements of this toolbox is multimodal data synchronization of at least 5 different signal types (EEG, kinematics, task-related events, physiological recordings (EOG, ECG), and audio) with Percept-LFP data. Following a precise protocol to deliver synchronization pulses, stimulation-induced artifacts were reproducible and reliable, resulting in an accurate automatic detection in most cases. Similar to earlier reports by Soh and colleagues^11^, we could demonstrate a temporal precision of 8 ms by relying on cardiac artifacts (a method that was more accessible in our setup than relying on Transcranial Magnetic Stimulation (TMS) pulses as they did). In their study, the temporal overlap of TMS pulses was only assessed 10 s after synchronization. While this is a good method to validate the accurate synchronization of data at the beginning of a recording, it doesn’t allow us to quantitatively assess the consistency and persistence of time-locking between external and Percept-LFP data across longer recording periods. A verification of the synchronization after 10–20 min of recording as proposed in our paradigm is highly valuable, as it is a more realistic duration for behavioral tasks with patients. This verification also revealed an important aspect that had not been addressed in implementations so far: Percept-LFP data can contain timeshift due to either packet loss (short interruptions of connection) or minor variations in the sampling frequency. A strategy to avoid interruptions is to always ensure proximity (<1 m) and absence of obstacles (even the patient’s own body) between the clinician tablet and the IPG during the recording and avoid the automatic sleep mode by touching regularly the tablet screen. The repetition of synchronization pulses at the end of each recording session allows to easily detect and correct timeshifts by adding NaN values during missing periods to compensate for packet loss and recomputing the *effective sampling frequency*.

Additionally, DBSsync offers a solution to another unmet need in Percept-LFP data preprocessing, which is a ready-to-use cardiac artifact removal pipeline. This pipeline demonstrated accurate and precise R-peak detection, a clear reduction in artifactual low-frequency power and a good recovery of beta oscillatory activity. The SVD method shows similar efficiency results as the template subtraction method but can better adapt to variations in the shape of the R-peak artifact and to various contamination levels. This method is the one we recommend using, therefore agreeing with recommendations from Stam and colleagues^13^.

With respect to the recording possibilities of the Percept^TM^ device, our paradigm can solely be used in the “BrainSense Streaming” mode, as it relies on the induction of stimulation artifacts. Thus, only one bipolar recording per hemisphere is possible with this synchronization technique. To perform synchronization with recordings from all contact pairs (within the so-called “Indefinite Streaming mode” of the Percept PC device), other methods need to be explored. In this regard, a recent paper proposed a different method for synchronization purposes, which relies on computer-driven artifact injection via triggered transcutaneous stimulation^14^. This method uses a NeuroOmega neurophysiology system to synchronize intracranial data with task event markers but has not yet been used to synchronize with other external data such as accelerometers or EEG.

Our method for synchronization has been developed based on the Percept^TM^ neurostimulator and a TMSi SAGA digital amplifier, but the concept can be adjusted to other neurostimulators with sensing capacity and other data recorders allowing for an external bipolar electrode (for example, magnetoencephalography recordings could also be in principle synchronized with Percept recordings if they can be recorded with an additional external bipolar electrode). Thanks to the versatility of the data types that can be streamed to LSL, our method allows for a high number of possibilities regarding the multimodal acquisition of data due to its compatibility with .XDF data formats. One important consideration to keep in mind when synchronizing cortical with subcortical data acquired through the Percept^TM^ neurostimulator is that phase estimation to perform coherence analysis between cortical and subcortical structures might be unreliable, specifically at higher frequencies, because of the inherent low sampling rate of such device and our achieved 8 ms precision in synchronizing data streams.

Overall, DBSsync is an open-source toolbox that doesn’t require a large setup or expensive equipment and enables intuitive use through a GUI. It is also versatile in terms of the various recording types that can be synchronized with Percept-LFP data, as well as in its preprocessing capacities to improve data quality and temporal precision. Our toolbox therefore provides the opportunity to synchronize intracranial brain activity along with externally recorded signals of interest (cortical EEG, accelerometers, behavioral task events, hand-tracking devices, etc.), for example in the framework of standardized behavioral tasks. This tool will help us realize new research protocols with multimodal recordings integrated to gain further insights into biomarkers, pathophysiology, brain networks and treatment effects in PD.

## Methods

### Patients

Twenty-five patients with PD and bilaterally implanted Medtronic SenSight leads in the STN connected to the Percept^TM^ neurostimulator (PC/RC, Medtronic, Minneapolis, MN, USA) were included in the study. Three different dataset types coming from three different studies were used to develop and validate the toolbox (Table 1).

Written informed consents were obtained from all patients, and the three studies were approved by the ethics committee at the Charité Universitätsmedizin Berlin (Dataset 1: EA2_256_20, Datasets 2 and 3: EA1/164/23) and conducted following the standards set by the Declaration of Helsinki.

### Datasets

Dataset 1 including *accelerometer data and LFP*: recordings were performed 3–24 months after DBS surgery as part of a long-term study investigating finger-tapping movements using accelerometers and intracranial LFP both with and without active DBS (*n* = 10 recording sessions).

Dataset 2, including *EEG and LFP during a behavioral task*: recordings were performed 7–38 months after DBS surgery as part of a study recording cortical and intracranial LFP during cued button presses both with and without active DBS (*n* = 19 recording sessions).

Dataset 3 including *hand movements in 3D-space, audio and LFP during a behavioral task*: recordings were performed 24–36 months after DBS surgery as part of a study recording kinematics, audio and intracranial LFP during a behavioral task both with and without active DBS (*n* = 5 recording sessions).

### Recording setup

For all datasets, intracranial brain activity was streamed from bilaterally implanted DBS-electrodes with a sampling frequency of 250 Hz via a Bluetooth connection to a tablet used for clinical programming. As external recording source for the synchronization artifact, a bipolar electrode with one contact placed close to the IPG and the other placed close to the IPG cable was connected to a digital amplifier (TMSi International, Oldenzaal, NL – TMSi SAGA) (Fig. 1a). Clinical evaluation of the stimulator placement and palpation of the IPG and cable were helpful to guide placement of electrode contacts. A wet wristband connected to the digital amplifier was also always worn by patients to ensure sufficient grounding and avoid amplifier saturation.

In dataset 1, two tri-axial accelerometers placed on both index fingers were recorded via the same digital amplifier. The output file of our digital amplifier was a .POLY5 file.

In dataset 2, the digital amplifier was also connected to a 32-channel EEG cap (BrainWave Cap Infinity – 32ch+gnd, Ag/AgCl – electrodes), an accelerometer placed on the finger used to press a response button during a behavioral task and two more bipolar electrodes used as electrooculogram (EOG) and ECG channels. Task events and signals from the digital amplifier were sent as two separate streams to the Lab Recorder application using the Lab Streaming Layer framework (LSL^15^,) which allowed for online automatic synchronization of these data streams. Using the Lab Recorder application, each recording resulted in a single output file in the eXtensible Data Format (.XDF) containing all synchronized external streams (Fig. 1b).

In dataset 3, the digital amplifier was solely used for recording the bipolar electrode used for synchronization (around the IPG). LSL was also used during the experiment and received four different streams: one from the digital amplifier containing the signal from the bipolar electrode, one from the behavioral task containing the event markers, one from the Ultraleap camera (Leap Motion Controller 2 (Ultraleap Ltd., Bristol, UK, 2023) or Stereo IR 170 Camera (Ultraleap Ltd., Bristol, UK, 2020) recording hand movements in 3D space and one from the audio recorder. These four streams resulted in a single .XDF file as output.

The output file of Percept-LFP recordings was a JavaScript Object Notation (.JSON) file, containing all recordings and metadata from an experimental session.

### Induction of DBS synchronization artifacts

We established a specific paradigm to induce stimulation artifacts in intracranial and external recordings for later data alignment. This paradigm consisted of two stimulation pulses at the beginning and end of each recording session (purple line in Fig. 1a). Specifically, the ramp option was deactivated to allow for large amplitude pulses and baseline recordings were started with DBS turned on and bilaterally set to 0 mA for 5 s. Two short pulses (approximately 2 s with an interval of 2 s each) of unilateral, high-frequency DBS at 1 mA amplitude were then delivered. DBS was then either kept at 0 mA (DBS-Off sessions, *n* = 17) or manually ramped up to the amplitude(s) tested in the study (DBS-On sessions, *n* = 17) (Table 1). Before ending each recording, DBS was set back to 0 mA bilaterally and two sequences of unilateral, high-frequency DBS with the same settings and in the same hemisphere as in the beginning were delivered. Importantly, patients were always made aware that the stimulation would be briefly switched from 0 to 1 mA for synchronization purposes, and that they could feel a little tingle. If discomfort is reported, we would recommend lowering the amplitude of the stimulation pulse to 0.5 mA or to the highest tolerated amplitude.

### Main interface and compatible file formats

DBSsync is a Python-based open-source toolbox developed to align intracranial Percept-LFP recordings and external recordings (available at: https://github.com/juliettevivien/DBSsync.git). The toolbox is designed with a GUI for easier use, and a user guide is available in the supplementary data (Supplementary Figs. 5–9 provide screenshots of the GUI). Within DBSsync, a Percept-LFP recording from one session (.JSON, .MAT or .FIF file) and the corresponding external recording from the same session (.XDF, .POLY5 or .FIF file) are loaded. If the external file is .XDF, the user is first asked to select which LSL stream contains the bipolar electrode used for synchronization. MAT file compatibility was implemented and designed for users who use the open-source Perceive toolbox (available at https://github.com/neuromodulation/perceive/) to extract each recording session as a single .MAT file. FIF files compatibility was added for both external and intracranial files to increase compatibility for MNE-python users (^16^; MNE-python) who might have their own preprocessing pipeline for .JSON files and/or different output formats for the external recordings.

### Synchronization of intracranial Percept-LFP and external recordings

In the time series of the external bipolar electrode recorded with the digital amplifier, the DBS artifact is detected as a steep and sustained decrease/increase (depending on electrode polarity) in signal amplitude after applying a high-pass filter (0.1 Hz) to detrend the data. The sample with the first highest amplitude change from baseline is selected as “start of the artifact” in the external channel (gray dashed line in Fig. 2b upper plot). In the intracranial recording, the DBS artifact is a sharp change in signal amplitude of the signal followed by a slow recovery (Fig. 2a lower plot). To identify at which point the sharp change occurs, a threshold window is computed based on the first 2 s of each intracranial recording. The last sample that lies within the value distribution of the window before crossing the threshold is detected as the start of the artifactual period (sample 0 in Fig. 2c). As the artifact in the Percept-LFP data spreads over several samples and doesn’t have a consistent shape across patients, we used a numerical labeling of each sample after this sharp change. To test which sample should be chosen as actual “start of the artifact”, 8 STN from independent sessions containing clear cardiac artifacts in both the Percept-LFP data and in an external ECG channel were used. Each sample was successively used for synchronization and the alignment of endogenous cardiac artifacts R-peaks in intracranial and external recordings was assessed 10 s later to reproduce the analysis performed with TMS pulses by Soh and colleagues^11^ for comparison. In all artifacts’ shape, the sample which overall provided the best temporal alignment of endogenous cardiac artifacts was the 4th sample after the sharp change (black sample in Fig. 2c) (mean time difference across 8 recording sessions ± std: 0 ± 4 ms, min: −6 ms, max +6 ms). Therefore, the 4th sample is defined as “start of the artifact” and is always used for synchronization in DBSsync (gray dashed line in Fig. 2b lower plot).

In the GUI, a plot shows the sample automatically chosen as the start of the artifact in both time series, allowing the user to decide whether the artifact has been properly detected. If the automatic detection method fails due to an unusual artifact shape or a baseline contaminated by other artifacts, the user can correct the DBS artifact detection and manually select the correct sample timepoint. Illustrations of how each artifact detection algorithm works are provided in Supplementary Fig. 1 (detection in intracranial recording) and Supplementary Fig. 2 (detection in external signal).

### Verification of the consistency of synchronization over time

In the timeshift window, the two synchronized channels can be plotted and overlapped to check the consistency of the synchronization over time using artifacts generated at the end of the recording sessions (Fig. 2d). DBSsync offers the possibility to compute the timeshift (i.e., the time-delay between these end-artifacts, see bottom plots in Fig. 2d). If the absolute value of the timeshift is high (>200 ms), it suggests that data loss happened in the Percept-LFP data, most times due to communication issues between the IPG and the clinician tablet. Such recordings should not be analyzed further before properly handling this data loss. If the Percept-LFP data was not loaded using the original .JSON file, consider using this method as it automatically corrects for packet loss. If the absolute value of the timeshift is below this value but still larger than 4 ms (temporal resolution of Percept device), it might reflect a slight variation in the sampling frequency of the Percept-LFP data from 250 Hz. This might not be considered relevant during short experiments but for longer recording sessions it produces inconsistent synchronization of the signals towards the end (up to 65 ms of timeshift was found in our datasets for a recording of ~11 min). Therefore, DBSsync also encompasses the possibility to compute the *effective sampling frequency* of the Percept-LFP data using the sampling frequency of the external digital amplifier as ground truth (usually, a much higher sampling frequency and data are not sent via Bluetooth, but via USB connection, which allows for higher accuracy in sampling frequency information). Illustration of how the *effective sampling frequency* is computed is provided in Supplementary Fig. 3. Once this *effective sampling frequency* is computed, it is automatically added to the Percept-LFP metadata and the synchronization artifact must be detected again in the LFP channel using this new sampling frequency for better accuracy. When computing the timeshift again, this results in a value close to 0 ms (Fig. 2d, right lower plot).

### Cardiac artifact removal from Percept-LFP data

DBSsync offers the possibility to clean Percept-LFP data from cardiac artifacts either after synchronization with external data or independently. The first step is to detect R-peaks in the LFP channel (Fig. 3a). The R-peak detection algorithm implemented in DBSsync is reproduced from procedures described by Stam and colleagues^13^. The contaminated channel can be used on its own or in combination with a synchronized external ECG channel recorded through the digital amplifier (recommended for better detection of R-peaks). A low-pass filter can be applied to the LFP data prior to cleaning, to remove potential stimulation artifacts and enhance signal-to-noise ratio (SNR).

If the Percept-LFP data is used alone, the detection algorithm will segment the signal into overlapping 1-s windows and search for negative and positive peaks in each window to get a first approximation of the R-peaks localization and their polarity. One-second epochs centered on these R-peaks are generated and averaged to create a first ECG template. The cross-correlation between this ECG template and the signal is calculated and R-peaks are identified if they exceed a 95th percentile threshold with a minimum distance of 0.5 s between peaks. A refined ECG template is computed, and a second-pass detection is performed in which the threshold can be modified by the user to refine the detection.

If an external ECG channel is synchronized and available to help detect R-peaks, this channel is band-pass filtered between 0.5 and 60 Hz to remove slow drifts and potential DBS artifacts and the algorithm searches for the timestamps of R-peaks in the ECG using a 95th percentile threshold and a minimum distance of 0.5 s between peaks. Corresponding R-peaks are searched in the LFP data by creating time windows from −80 ms to +80 ms around each R-peak timepoint as detected in the ECG (the algorithm searches for local minima/maxima values in each window in the LFP data). These two detection methods are reproduced from the description provided by Stam and colleagues^13^. DBSsync offers the possibility to manually override some of the parameters for better control of R-peak detection (e.g., wrong polarity detected, artifactual periods to avoid, percentile threshold to use).

The second step is to clean the signal from ECG artifacts based on the detected R-peaks. The methods implemented in DBSsync are also adapted from the paper published by Stam and colleagues^13^ in which they extensively describe three different pipelines for cardiac artifact removal in Percept-LFP recordings. Two of these three methods are available in DBSsync to dampen the cardiac artifact: (1) a template subtraction method, in which an average template of the QRS complex is generated, which is then fitted and subtracted to each cardiac artifact individually, and (2) a singular value decomposition (SVD) method, in which the cardiac artifact is reconstructed at each R-peak based on the first “k” components of the SVD, fitted and subtracted separately, to account for differences in artifact shape. The number of “k” SVD components to use for the reconstruction is set by the user, based on the shape (as described in^13^) and explained variance of each component.

DBSsync automatically displays four output plots to assess the quality of cardiac artifact removal: (1) detected R-peaks (Fig. 3a), (2) ECG artifact shape (average template across epochs or average reconstruction using SVD components), (3) an overlap of the raw and cleaned channel (Fig. 3b, left) and (4) an overlap of the power spectrum of the raw and cleaned data (Fig. 3b, right). Once cardiac artifacts are properly removed, the user can choose to replace the original channel with the cleaned one. The original channels will still be saved in the output file but labeled as “RAW”.

### Validation of cardiac artifact removal

Across 68 STN recorded, 30 contained visible cardiac artifacts (Table 1). R-peak detection was performed in these STN recordings without using any external ECG channel and the accuracy of the detection was assessed by counting true positive, false positive and missed peaks over 1 min of recording (Fig. 3a). The minute chosen for testing R-peak detection accuracy was always from 80 to 140 s, as it was the first common minute free of synchronization pulses in all recordings. When available, this was performed again using an external ECG channel to help R-peaks detection. The best detection was kept for further cleaning of the channel. In some cases, the “manual override” option was used to correct for ECG artifact polarity (5 cases) or the start/end of detection (to avoid stimulation pulses, which can bias the ECG artifact shape if included in the template).

Power spectra of raw and cleaned channels were computed using Welch’s method with a 1 s window and 50% overlap. These power spectra were used to determine ECG suppression ratio and beta peak recovery achieved by each method. These metrics were chosen and adapted from the thesis of Silvi L.^17^. The ECG suppression ratio measures power attenuation with higher values indicating greater cardiac artifact removal. It is calculated as:

1 $$ECG\,suppression\,ratio=10lo{g}_{10}\left(\frac{{P}_{ECG,raw}}{{P}_{ECG,cleaned}}\right)$$

with *P* the average power in the ECG-specific frequency band defined from 0.5 Hz to 40 Hz. Beta peak recovery measures the preservation of oscillatory activity in the beta frequency band (13–35 Hz) with values close to 1 indicating optimal preservation. It is calculated as:

2 $$Beta\,peak\,recovery=\frac{{Peak\,prominence}_{cleaned}}{{Peak\,prominence}_{raw}}$$

The peak prominence is calculated relative to the surrounding spectral baseline within an extended frequency window (±5 Hz around peak frequency). In our datasets, 7 STN contained a prominent beta peak and were used for this analysis. For statistical assessment of each metric, a Wilcoxon signed-rank test was performed.

To further validate cleaning methods, a synthetic signal resembling LFP signal was generated. This signal was composed of a 6 Hz sine wave (to mimic theta oscillation) and a 23 Hz sine wave (to mimic beta oscillation) with 1/F noise and high pass filtered at 0.01 Hz to simulate the effect of Percept’s hardware filter. An externally recorded ECG signal was used to contaminate the synthetic LFP signal with different levels of SNR, ranging from −10 dB to +10 dB (examples of contaminated signals are provided in Supplementary Fig. 4a). Power spectra of ground truth synthetic signals and cleaned signals were computed using Welch’s method with a 1 s window and 50% overlap for each SNR contamination level (Supplementary Fig. 4b). For each cleaning method and contamination level, four measures were calculated: SNR improvement, correlation with ground truth, BPP and TPP.

#### Signal-to-noise ratio (SNR) improvement

The SNR between the ground-truth (uncontaminated) signal *x*(*t*) and a tested signal *y*(*t*) (representing either the contaminated synthetic signal or the signal cleaned by one of the methods) was computed in decibels as

3 $${\rm{SNR}}=10{\log }_{10}\left(\frac{{P}_{\mathrm{signal}}}{{P}_{\mathrm{noise}}}\right)$$

where the signal power is

4 $${P}_{\mathrm{signal}}=\frac{1}{N}{\sum }_{t=1}^{N}x{\left(t\right)}^{2}$$

and the noise power is defined from the residuals

5 $$n\left(t\right)=x\left(t\right)-{\rm{y}}\left(t\right)$$

6 $${P}_{{\rm{noise}}}=\frac{1}{N}{\sum }_{t=1}^{N}n{\left(t\right)}^{2}$$

SNR improvement introduced by each artifact removal method was defined as the difference between the SNR of the cleaned signal (SNR_cleaned_) and the SNR of the contaminated signal (SNR_contaminated_):

7 $${\rm{SNR\; improvement}}={{\rm{SNR}}}_{\mathrm{cleaned}}-{{\rm{SNR}}}_{\mathrm{contaminated}}$$

#### Correlation with ground-truth signal

Similarity between the cleaned signal and the ground-truth (uncontaminated) signal was quantified using the Pearson correlation coefficient.

#### Beta/theta power preservation (BPP/TPP)

To evaluate whether artifact-removal methods preserved oscillatory activity of interest, we computed BPP and TPP by comparing the power in the beta/theta frequency bands between the cleaned signals and the ground-truth (uncontaminated) signal. Power spectral density (PSD) was estimated using Welch’s method with a sampling frequency of 250 Hz, a window length of *N* = 250 samples, and 50% overlap. Beta band was defined as 21–25 Hz and theta band was defined as 4–8 Hz, respectively centered around the synthetic 23 Hz and 6 Hz oscillations embedded in the simulated LFP.

Beta band power was calculated by summing the PSD values within the selected frequency range:

8 $${P}_{\beta }={\sum }_{f\in \left[\mathrm{21,25}\right]}{PSD}\left(f\right)$$

BPP/TPP was then defined as the ratio between the power of the cleaned signal and that of the ground-truth (uncontaminated) signal:

9 $$BPP=\frac{{P}_{\beta }^{{\mathrm{cleaned}}}}{{P}_{\beta }^{uncontaminated}}$$

Similar formulas were used for theta band power. BPP/TPP = 1 indicates perfect preservation of band power, whereas values below or above 1 indicate underestimation or overestimation of band power after processing.

### Saving synchronized and/or preprocessed recordings

The saving option can be set by the user in the config file before starting DBSsync. Available saving options are .SET, .FIF, .MAT or .PKL. In the case of external .XDF files, users should be cautious of what type of stream was contained in it: if it only contained continuous streams and marker streams, the synchronized/cleaned data can be saved in .SET, .MAT or .FIF format. However, if the .XDF file contained discontinuous streams (i.e., without a defined sampling frequency but only discrete data points) the synchronized data must be saved as .PKL (as the other formats only allow for one common sampling frequency for all channels).

## Supplementary information

Supplementary Information

## Data Availability

Data are available conditionally through data-sharing agreements in accordance with data privacy statements signed by the patients within the legal framework of the General Data Protection Regulation of the European Union, within a time frame of 6 months. Requests should be directed to AAK (andrea.kuehn@charite.de) or the Open Data officer (OpenData-Neuromodulation@charite.de).

## Code availability

The code of the toolbox is publicly available in a Github repository and can be accessed via this link: https://github.com/juliettevivien/DBSsync.
