# Supplementary material for: DBSsync: combining intracranial and multimodal data to investigate new biomarkers in Parkinson’s disease
Source: NPJ Parkinsons Dis. 2026 Jun 19;12:151. doi: 10.1038/s41531-026-01439-z (PMC13279824; doi:10.1038/s41531-026-01439-z)
Supplement: Supplementary file 1 — Supplementary Information [file 41531_2026_1439_MOESM1_ESM.pdf]

Supplementary figures:

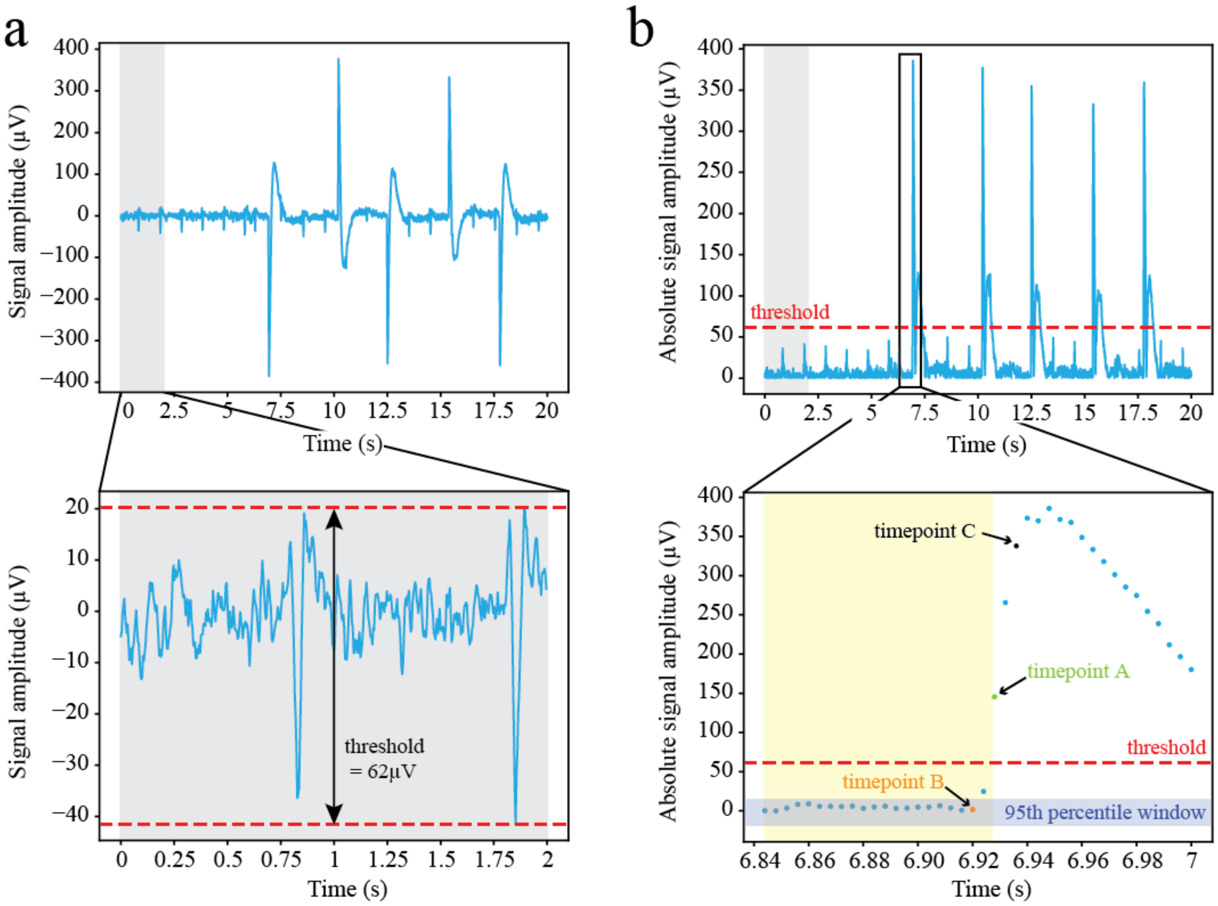

Supplementary figure 1: description of the algorithm to detect the synchronization artifact in the intracranial channel.

a) A threshold is computed based on a 2-second baseline window (grey shaded area): the threshold is the maximum peak-to-peak amplitude.

b) Data is transformed into absolute values (this way, artifact polarity does not matter) and the first moment where data crosses the threshold is detected (timepoint A). In the time window from the beginning of the recording to timepoint A (yellow shaded area), the last sample lying in the 95th percentile of the baseline value distribution (timepoint B) is detected. From timepoint B, the 4th sample is selected as “start of the artifact” = timepoint C.

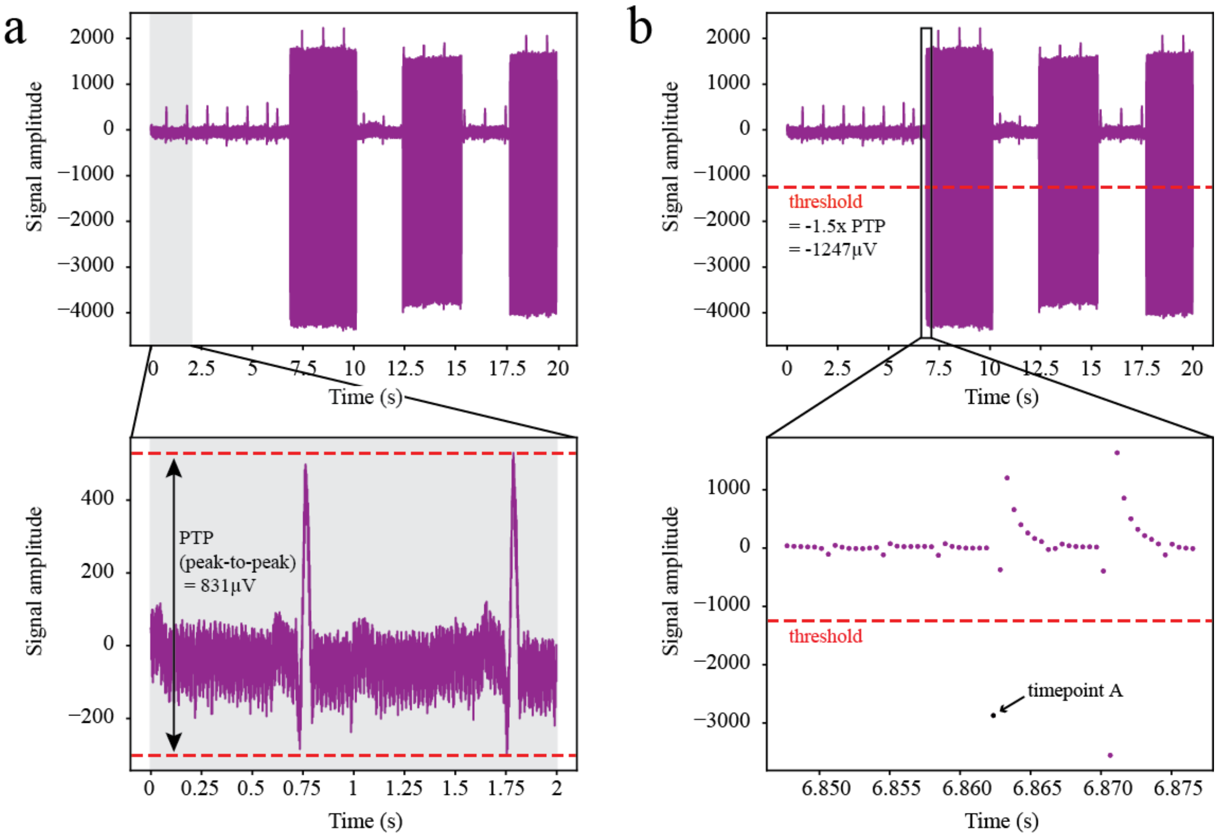

Supplementary figure 2: description of the algorithm to detect the synchronization artifact in the external channel.

The polarity of the artifact is assessed: if positive values are higher than absolute negative values, artifacts are positive and the signal is multiplied by -1 to invert it (algorithm looks for negative peaks). In this example, the signal is already well oriented.

a) A threshold is computed based on a 2-second baseline (grey shaded area): the threshold is the maximum peak-to-peak (PTP) amplitude multiplied by -1.5 (to detect only values that are at least 50% lower than threshold.

b) The first sample crossing the threshold AND with a smaller value than BOTH its neighboring samples is selected as “start of the artifact”.

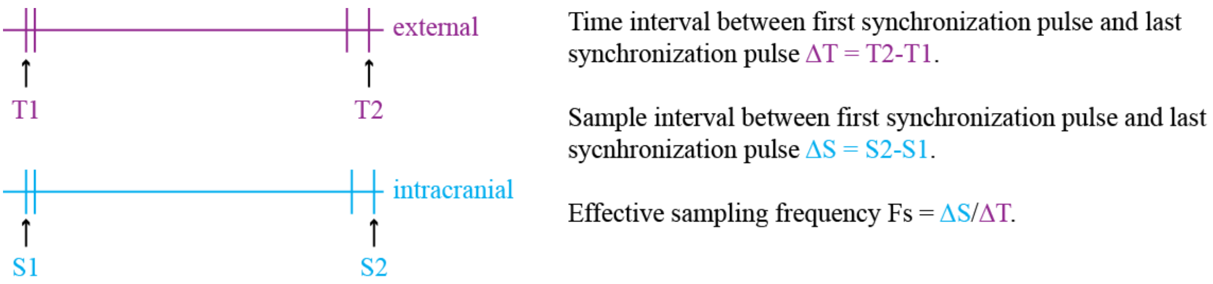

Supplementary figure 3: description of the effective sampling frequency calculation.

The user is asked to detect the first and the last synchronization pulses in both the intracranial and the external recording. Because the external sampling frequency is used as ground truth, it is possible to determine exactly how much time separates the last from the first synchronization pulse: $\Delta T = T2-T1$. The algorithm also calculates how many samples in the intracranial recording separate the first from the last synchronization pulses: $\Delta S = S2-S1$. These two values are then used to infer the effective sampling frequency of the intracranial recording: $Fs = \Delta S/\Delta T$.

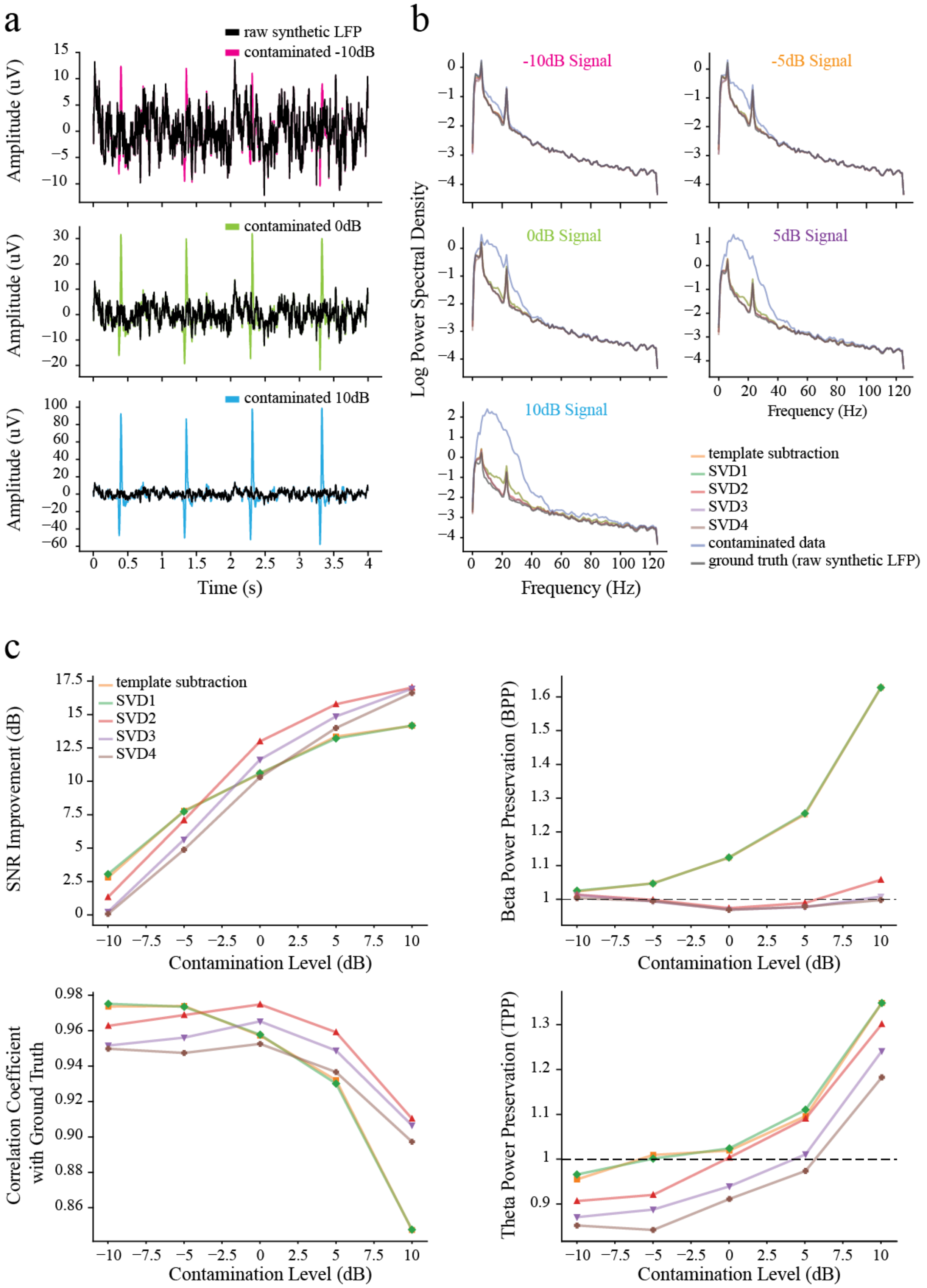

Supplementary figure 4: cardiac artifact removal on synthetic signals contaminated at different signal-to-noise ratios.

A synthetic signal containing a 6Hz (theta) sine wave, a 23Hz (beta) sine wave and 1/F noise was generated. Using an ECG externally recorded, synthetic signals were contaminated at different predetermined Signal-to-Noise Ratios (SNR), ranging from -10dB to +10dB (decibels).

a) Example signals with -10dB, 0dB and +10dB contamination.

b) Power spectrums at each SNR level with different cleaning methods applied.

c) Performances of each cleaning method on SNR improvement (top left), correlation of cleaned signal with original signal (bottom left), beta power preservation (top right) and theta power preservation (bottom right).

SVDx: Singular Value Decomposition using x components

Documentation DBSsync

Open-source Python GUI for preprocessing and synchronizing intracranial data from sensing-enabled DBS electrodes with external data

General Information

DBSsync is a Python Graphical User Interface (GUI) for synchronizing intracranial data from Deep Brain Stimulation (DBS) electrodes with external data. It also contains preprocessing options to perform cardiac artifact removal in these recordings. It is designed to be user-friendly and to facilitate the synchronization process. The GUI is built using the PyQt5 library and is compatible with Windows, MacOS, and Linux operating systems. It is open-source and can be freely downloaded and modified by users.

Supported data formats:

DBSsync synchronizes data from DBS electrodes ("intracranial data") with external data, and/or performs ECG cleaning on intracranial channels.
The supported data formats are:

- For the intracranial data: .json, .mat, .fif
  - .JSON files is the native format of files recovered from clinicial recording tablets after a recording session. It contains all the session's data and metadata.
  - The GUI can also be used on files already preprocessed with the perceive toolbox. This toolbox loads the .JSON file recovered from the clinician recording tablet after a recording session, and generates BIDS-inspired subject and session folders with the ieeg format specifier. All time series data are being exported as FieldTrip '.mat' files.
  - .FIF files compatibility has been implemented for loading datasets already preprocessed and saved, using MNE-python for example.

- For the external data: .xdf, .fif, .Poly5.
  - The .xdf format is the output of Lab Streaming Layer (LSL)

- The .fif format is the output of common MEG/EEG devices and of MNE-python saved datasets
- The .Poly5 format is the output of TMSi data recorder

Supported saving formats after synchronization/preprocessing:

The synchronized/preprocessed data can be saved in the following formats: .set, .fif, .mat, .pkl

- If the external dataset was a file with a unique sampling frequency (e.g. .xdf file containing an EEG recording and task events), or if only intracranial data was loaded for cardiac artifact removal:
  - .set
  - .fif
  - .pkl
  - .mat
- If the external dataset has discontinuous data points, without a unifying sampling frequency:
  - .pkl

Installation

1 Clone the repository: `git clone https://github.com/juliettevivien/DBSsync`

2 Navigate to the local version of DBSsync and create the virtual environment

- Manually, using anaconda prompt: follow the commands from Create virtual env.txt
- Using pip: `pip install -r requirements.txt`
- Using Conda: `conda create --name  --file requirements.txt`

3 Activate the virtual environment using the command: `conda activate env_name`

4 Run the GUI using the command: `python DBSsync_main.py`

Usage

1 **Synchronization of intracranial data with external data**

1. Open the GUI by running the command: `python DBSsync_main.py`

2. Load the intracranial data by clicking on the "Load Intracranial file" button.

3. Load the external data by clicking on the "Load External file" button. If the external data is in .xdf format, DBSsync GUI will automatically detect the streams contained in the file and a pop up window will ask to select the stream containing the bipolar channel used for synchronization.

4. For each data type, select the channel containing the synchronization artifacts. See the synchronization protocol for more information about how to create the artifacts during the recording. You can plot the channel to make sure you see the artifacts before automatically detecting them.

5. Detect the first artifact, either by using the automatic or the manual method. See on the Supplementary figure 5 below which sample should be detected as the start of the artifact for a reliable synchronization.

Supplementary figure 5: Screenshot of the GUI's Home Page.

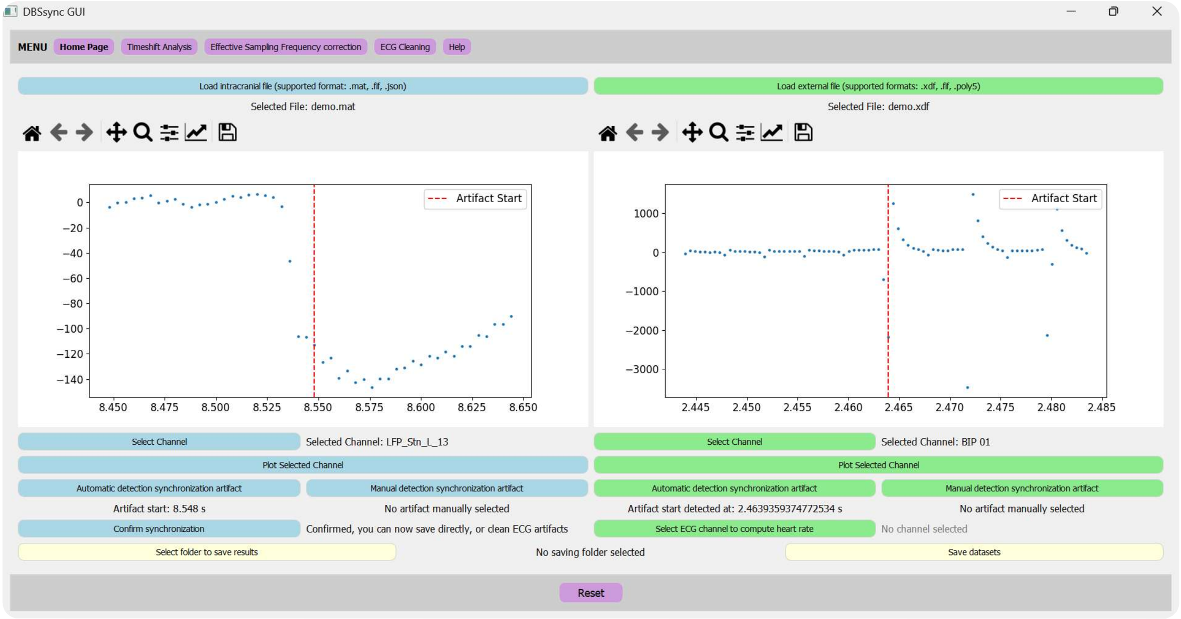

On the left side is the panel about the intracranial recording, on the right side is the panel for the external recording. In this example, artifacts were selected automatically by DBSsync and the selection is correct:

- Intracranial data: the point selected as the start of the artifact should always be the 4th sample after the last one before the amplitude drops (or increases when the polarity of the signal is reversed compared to this example)

- External data: the point selected as the start of the artifact shoud always be the first lowest sample after the amplitude changes (or the first highest sample when the polarity of the signal is reversed compared to this example)

2 Timeshift Analysis

Check the synchronization consistency over time: assess the "timeshift". Plot the synchronized data together and verify if the last artifact is also aligned. Checking the timeshift is an important step to ensure that there was no data loss during the recording and no deviation from the 250Hz sampling frequency of the Percept device.

- If the absolute value of the timeshift is higher than 200ms, it might be a good idea to check for packet loss in the intracranial data. Try loading directly the .JSON file if this wasn't the case yet, as DBSsync automatically detects and corrects for packet loss.
- If the absolute value of the timeshift is smaller but still higher than 10ms (see Supplementary figure 6 for an example), adjust effective sampling frequency of the intracranial data.
- IMPORTANT: sampling frequency SHOULD NOT be adjusted if the timeshift is >200ms because this is a sign of packet loss, not of inaccurate sampling frequency. Missing packets should first be detected and replaced by NaNs, before synchronizing again and correcting the sampling frequency.

Supplementary figure 6: Screenshot of the GUI's Timeshift Analysis page.

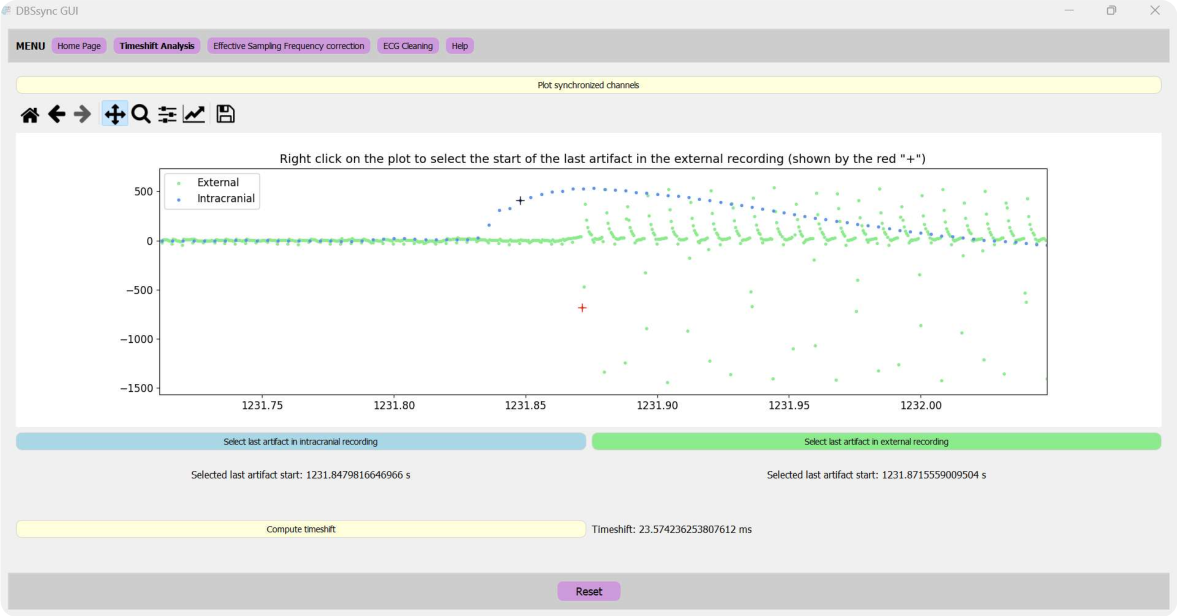

In this example, artifacts were manually selected in the intracranial and external recordings. The timeshift is calculated as the difference between the last artifact in the intracranial data and the last artifact in the external data. In this example, the timeshift is 24ms, which means that the sampling frequency of the intracranial data should be corrected before saving.

3 Effective Sampling Frequency Correction

To calculate the effective sampling frequency of the intracranial data, click on "Effective sampling frequency correction". In this window, both the intracranial and the external channels are plotted (see Supplementary figure 7 below for an example). The first and last artifacts should be manually detected in each channel (see Supplementary figure 8 below for an example). The effective sampling frequency is calculated as the number of samples between the two artifacts (in the intracranial signal) divided by the time difference between the last and the first artifact (in the external signal). Once calculated, this "effective" sampling frequency is automatically applied to the intracranial file. Please reselect the first artifact in the intracranial data in the home page to apply the correction before saving or proceeding to ECG artifact cleaning. The corrected data can also be plotted again in the timeshift analysis window to check if the synchronization is now correct (timeshift should be close to 0 after correction).

Supplementary figure 7: Screenshot of the GUI's Effective Sampling frequency window.

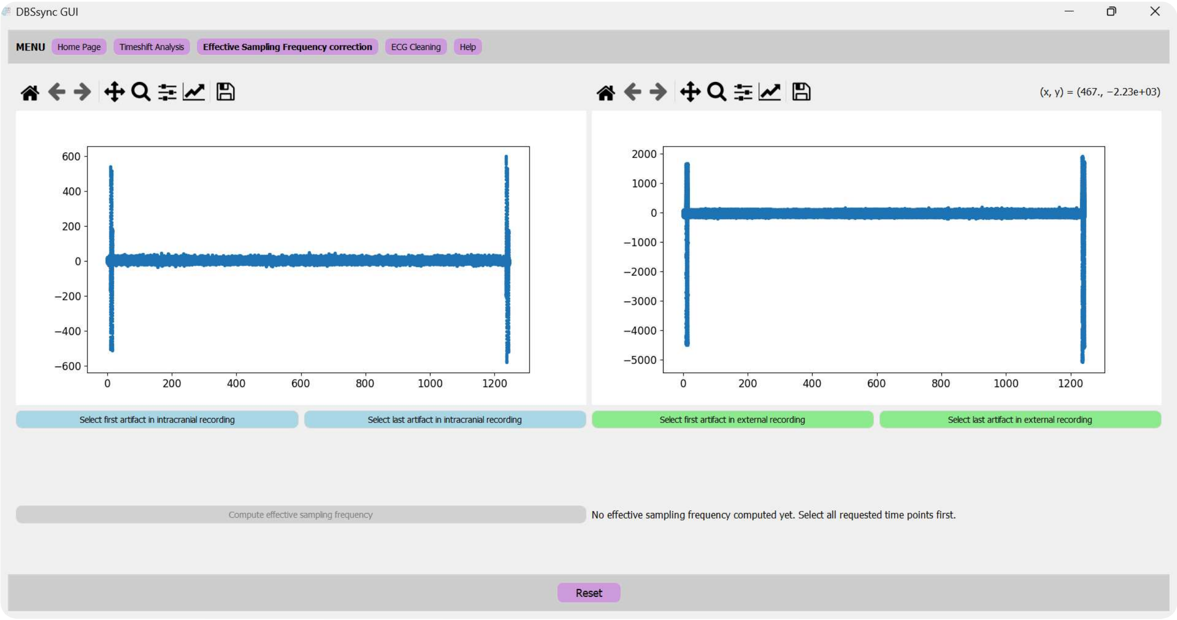

On the left side is the panel about the intracranial recording, on the right side is the panel for the external recording. The signal of each channel is automatically plotted as a scatter plot to facilitate manual selection of the first and last artifact in each channel.

Supplementary figure 8: Calculation of the effective sampling frequency of the intracranial signal.

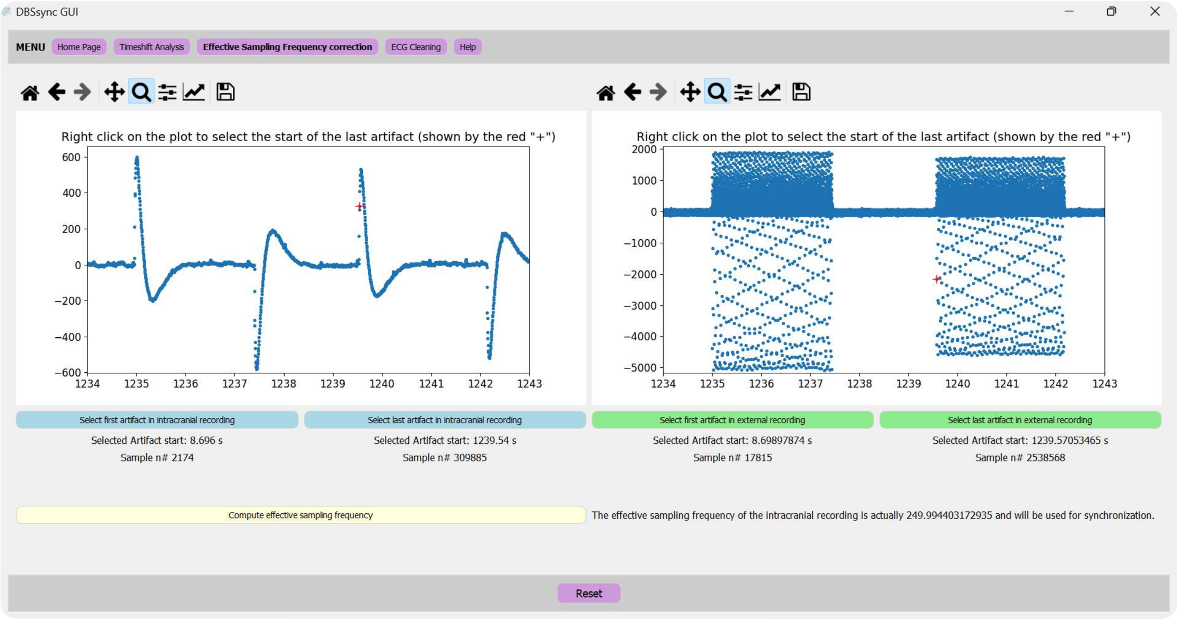

Red crosses highlight manual selection of the last artifact in each recording: it should correspond to the beginning of the last stimulation pulse in both channels. The first artifacts were also previously selected. The effective sampling frequency is calculated as the number of samples between the two artifacts in the intracranial signal divided by the time difference between the last and the first artifact in the external signal. In this example, the effective sampling frequency is 249.994403172935Hz.

4 ECG Artifact Cleaning

Optional: clean ECG artifacts in the intracranial data.

Supplementary figure 9: Screenshot of the GUI's ECG cleaning window.

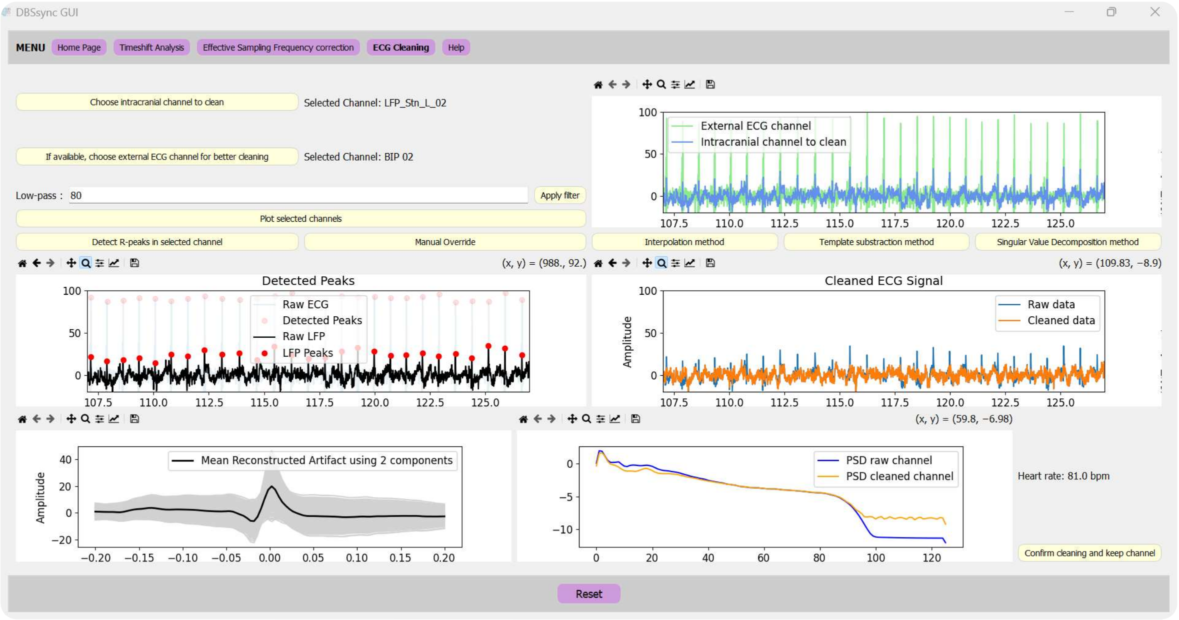

In this example the ECG artifacts were detected in the intracranial data using an external ECG channel to help detection.

- The first plot displays the detected R-peaks in both the external channel (light traces) and the intracranial channel (dark traces).
- The second plot shows the average shape of the ECG artifact, reconstructed using 4 components from the singular value decomposition method.
- The third plot shows an overlap of the raw and cleaned channel after applying SVD4 (SVD method using 4 components).
- The fourth plot shows an overlap of the power spectrum from the raw and cleaned channels after applying SVD4.

The heart rate is also automatically computed and displayed in the ECG cleaning window.
If the cleaning is satisfactory, you can click on "Confirm cleaning and keep channel".

To clean the ECG artifacts in the intracranial data, click on "ECG cleaning". In this window, an intracranial channel containing ECG artifacts can be select and plotted to assess the presence of ECG artifacts. A low-pass filter can be applied to the intracranial data to remove stimulation artifacts before applying the ECG cleaning algorithm. If available, a synchronized external ECG channel can also be selected to help for R-peaks detection. Click on "Detect R-peaks". Detected R-peaks are automatically plotted below, and the user can check if the detection is correct. If the detection is not correct, some parameters can be manually overridden in the Manual Override section, before clicking on "Detect R-peaks" again:

- **R-peak polarity** If the polarity of R-peaks is wrongly detected in intracranial channel, update it manually.
- **Start and end of detection:** DBSsync should avoid stimulation pulses periods when detecting R-peaks, to create a reliable template. If some R-peaks are detected in stimulation pulses, please update these parameters to make sure they are not detected.
- **Periods to avoid:** same as for stimulation pulses, large artifacts should also be avoided when possible to get a reliable template. Enter here time periods to be avoided as tuples, e.g. [(2.2, 3), (80.4, 82.7)].

Once R-peaks are correctly detected, one of the three cleaning methods available

in the toolbox can be used:

- **Interpolation method:** this method performs a linear interpolation of R-peaks in the intracranial data.
- **Template subtraction:** this method creates an average template of the QRS complex based on detected R-peaks. At each R-peak, the template is fitted using linear fit, tails are equalized and the tempalte is substracted from the corresponding R-peak in the intracranial data.
- **Singular value decomposition:** this method creates a matrix of all QRS complexes around detected R-peaks. Using singular value decomposition, it plots the first 4 components with their explained variance and prompts the user to choose how many components to keep for the reconstruction of the QRS complex. At the level of each R-peak, the chosen components are used to reconstruct the QRS complex around that specific R-peak and the reconstructed QRS complex is then fitted using linear fit, the tails are equalized and the resulting QRS complex is substracted from the corresponding R-peak in the intracranial data.

This methods are reproduced from the paper of Stam and colleagues (2023):
M.J. Stam, B.C.M. van Wijk, P. Sharma, M. Beudel, D.A. Piña-Fuentes, R.M.A. de Bie, P.R. Schuurman, W.-J. Neumann, A.W.G. Buijink, A comparison of methods to suppress electrocardiographic artifacts in local field potential recordings, Clinical Neurophysiology, Volume 146, 2023, Pages 147-161, ISSN 1388-2457, https://doi.org/10.1016/j.clinph.2022.11.011.

Three automatic plots will be generated:

- the first plot shows the average template used for the cleaning
- the second plot shows an overlap of the raw and cleaned channels
- the third plot shows an overlap of the power spectrum from the raw and cleaned channels

5 Saving synchronized data

Choose the fileformat in which you want to save the synchronized data, and a folder to save the results. Available saving options are .set, .fif, .mat, .pkl. IMPORTANT: if your xdf file contains multiple streams with various sampling frequencies, do not save in .SET format, because the .SET format does not support multiple sampling frequencies. In this case, you can only save in .pkl format. You can choose to save all recordings/streams in a single pkl file, or to save each recording/stream in a separate pkl file.

Synchronization Protocol

The synchronization protocol is a set of guidelines to create reliable synchronization artifacts during the recording. These artifacts are used later on to synchronize intracranial data with external data thanks to this GUI.

Steps:

1. **Prepare the recording:** Setup the BrainSense Streaming mode. Deactivate the ramp option of the stimulation on the clinician recording tablet. Keep the stimulation ON, but set it at 0mA bilaterally.

2. **Start the recording:** Start the recording of the intracranial data and the external data.

3. **Generate the artifacts (2 times):** In the clinician recording tablet, increase the stimulation amplitude to 1mA unilaterally in one step. This will generate a clear artifact in the intracranial data and in the bipolar electrode channel placed in proximity to the implantable pulse generator, which will be used for synchronization.

4. **Perform your recording** After generating the artifact, decrease the stimulation amplitude to 0mA bilaterally if you are recording in DBS OFF, or slowly ramp up the stimulation back to the clinical settings if recording in DBS ON.

5. **Repeat the artifact at the end:** Repeat the stimulation pulses at the end, before stopping the streaming. (Do not forget to put both hemispheres at 0mA before doing the pulses, and do the pulses ON THE SAME SIDE as the first artifact).

6. **Stop the recording:** Stop the recording of the intracranial data and the external data.
